# Study on a Two-Stage Computational Model for the Mechanical Behavior of Metal Ring Nets in Flexible Rockfall Barriers

**DOI:** 10.3390/ma19194128

**Published:** 2026-09-27

**Authors:** Yana Zhao, Chang Yang, Tao Wei, Ahad Amini Pishro, Lili Zhang

**Affiliations:** 1 School of Civil Engineering, Sichuan University of Science and Engineering, Zigong 643000, China; zhao_yana@suse.edu.cn (Y.Z.); gotowt@126.com (T.W.); ahad.ap@suse.edu.cn (A.A.P.); zhanglili@suse.edu.cn (L.Z.); 2 College of Architecture and Urban-Rural Planning, Sichuan Agricultural University, Chengdu 611830, China

**Keywords:** flexible rockfall barrier, metal ring net, two-stage computational model, tensile and puncture tests, full-scale impact test, energy dissipation

## Abstract

Metal ring nets are the core interception components of flexible rockfall barriers, and the accurate characterization of their mechanical behavior is essential for the safety assessment of the whole system. To overcome the limitations of existing computational models in reflecting the large-deformation states of the nets and the activation of energy dissipators, systematic uniaxial tension tests and puncture tests were first carried out on five specifications of ring nets (R8 to R19, 30 specimens in total). The results reveal a two-stage deformation feature (an initial low-force tensioning stage followed by a stiffness-hardening rapid-rise stage) and a parallel-superposition load-carrying mechanism under both loading cases. The experimental data were then normalized by dividing the force by the number of steel wires and non-dimensionalizing the displacement, on the basis of which a unified piecewise two-stage constitutive model, consisting of a linear tensioning segment and a power-hardening segment, was established to describe the response up to the peak point; the tensile and puncture cases share the same model form and are distinguished only by different parameters (fitting coefficient of determination *R*^2^ not lower than 0.97, with mean values of 0.98 and 0.983, respectively). A full-scale numerical model of the overall structure was subsequently established using LS-DYNA, in which the two side spans adopt the tensile constitutive model and the middle impact span adopts the puncture constitutive model. The model was verified against a 1500 kJ full-scale impact test. The computed time histories of the support-rope forces, the activation states of the energy dissipators (pressure-relief rings), the maximum interception deformation (computed 8.23 m versus measured 8.8 m, with a relative error of about 6.5%) and the energy-dissipation distribution of the pressure-relief rings (average relative error of about 5.2%) all agreed well with the experimental results. The proposed two-stage computational model can therefore accurately reflect the force and deformation states of the ring nets in the actual structure and correctly describe the transmission of the impact force and the activation of the energy dissipators, providing a reliable theoretical basis for the refined design of flexible rockfall barriers.

## 1. Introduction

Flexible rockfall barriers are widely applied as protection against rockfalls, slope debris flows, gully debris flows, and tunnel rockbursts, dissipating the enormous impact kinetic energy at the instant of interception through their flexible large-deformation energy dissipation mechanism [1,2,3,4]. The flexible metal net is the interception member that directly sustains the impact to the structure; it fully slides while undergoing large deformation, activating the energy dissipators to deform and dissipate energy, and provides more than 70% of the overall structural deformation, being one of the most widely used core members of such structures (Figure 1). The metal ring net is fabricated by winding high-strength steel wires into rings, which are interconnected by a certain interlocking pattern to form the net panel; under impact, it undergoes adaptive deformation and sliding, exhibiting an extremely high deformation capacity. However, the loose configuration and adaptive deformation also give rise to pronounced multi-flexible-body nonlinear dynamic characteristics and a non-uniform force-flow distribution, such that the accurate calculation of its mechanical behavior still faces many challenges [5].

The research on the mechanical models of ring nets has evolved from simple to complex. Early studies were mostly based on linear elastic theory, simplifying the net into a homogeneous material or discrete elements [6]; for example, the zoned equivalent model proposed in Ref. [7] combined ring tension tests with numerical simulation to establish the load–displacement relationship of the net under typical deformation states. In recent years, scholars have gradually introduced nonlinear mechanical theories to improve the model accuracy [8,9,10]. Refs. [11,12] revealed the three-stage working characteristics of ring nets through puncture tests and proposed an analytical method for the tensile deformation. Studies based on tension and puncture tests have also been deepened progressively, providing data support for the mechanical modeling [11,12,13,14,15]. Nevertheless, the existing models still have limitations in comprehensively considering both the deformation of the net itself and the activation degree of the energy dissipators.

A review of the existing studies shows that current theoretical studies and numerical simulations mostly simplify the ring net into an isotropic continuum membrane or an orthogonal bar system to reduce the computational complexity. However, such over-simplification can hardly reflect the funnel-shaped deformation mechanism of the net under localized impact, nor accurately simulate its large-deformation behavior, resulting in the impact load failing to be effectively transmitted to the ends of the support ropes [16,17]. Recently, to overcome the limitations of the off-the-shelf LS-DYNA modeling, several advanced computational strategies have been developed for the refined simulation of flexible protective structures. Qi et al. [18] established a numerical modeling approach for flexible rockfall protection barriers based on failure modes, and Xia et al. [19] studied the numerical model and dynamic response of ring nets in flexible rockfall barriers. Singer et al. [20] proposed a partitioned MPM–FEM coupling approach that captures the large-deformation response of mass-movement hazards impacting flexible barriers. Debelak et al. [21] established a finite-element model of a flexible steel ring-net debris-flow barrier, reproducing its impact loading and deformation processes. Liu et al. [22] quantified the impact of a debris avalanche against a flexible barrier through coupled DEM–FEM analyses, revealing the load-transfer mechanism between the impacting mass and the net. These advanced methods improve the physical consistency of the simulation; however, they mainly address the structural-level response and still rely on refined discretization, which is computationally demanding and less convenient for parametric studies. In contrast, the equivalent-constitutive strategy adopted in the present work provides an efficient alternative that captures both the deformation of the net itself and the activation of the energy dissipators with a compact mechanical model.

Related studies indicate that the energy dissipators provide more than 50% of the energy dissipation of the protection system and are its most critical energy-dissipating components [23,24]. When the deformation capacity of the net model is limited, the support ropes are blocked by friction at the steel posts, and the tension transmitted to the energy dissipators is insufficient, causing the energy dissipators to fail to activate, and in extreme cases, this even causes systemic failure of the support ropes due to a sudden tension surge (peak exceeding 360 kN). Moreover, the ring net exhibits evident staged characteristics (elastic and hardening stages) during loading, and the mechanical responses of nets of different specifications differ greatly; the existing models can hardly take into account both the deformation state of the net itself and the activation degree of the energy dissipators.

In view of the above problems, this paper aims to establish an improved two-stage computational model that can more realistically reflect the mechanical behavior of ring nets and their synergistic working mechanism with energy dissipators. First, systematic uniaxial tension and puncture tests were carried out on ring nets of five specifications from R8 to R19 to obtain their mechanical response parameters under different loading states and to reveal the deformation mechanism and failure modes. Then, based on the test data, an improved multi-stage computational model accounting for material nonlinearity and structural geometric characteristics was constructed, focusing on the description of the stiffness hardening of the net in the large-deformation stage. Finally, the actual rockfall impact condition was simulated by a full-scale impact test, and the measured net deformation and energy-dissipator activation data were compared with the model predictions, providing a reliable theoretical basis for the refined design and safety assessment of flexible protection nets.

## 2. Uniaxial Tension and Puncture Test Study of Metal Ring Nets

### 2.1. Test Scheme Design

To comprehensively elucidate the mechanical properties of metal ring nets, this study formulated a test scheme covering multiple loading states and net specifications, including uniaxial tension tests and puncture tests [11,15]. The uniaxial tension test aims to reveal the mechanical behavior of the ring net under axial loading, while the puncture test focuses on the penetration resistance and failure modes of the ring net under localized impact loading, with the specimen specifications covering the commonly used range. The systematically obtained mechanical properties provide basic data for establishing the subsequent improved computational model. The test instruments are shown in Figure 2.

Considering the specification range of commonly used metal ring nets, practical engineering applications, and the objective requirements of the theoretical analysis for data volume, five specifications of ring-net specimens were selected (Table 1), and three specimens of each specification were subjected to the tension and puncture tests, respectively. All the ring nets tested were fabricated from high-strength steel wires with a nominal strength of 1770 MPa. The specimens were prepared, and the tests were carried out in accordance with the Chinese transportation industry standard JT/T 1328-2020 “Flexible Protection Net System of Slope” [25].

The uniaxial tension tests were carried out using a commercial hydraulic tension testing machine with self-made tension fixtures, with a maximum loading capacity of 1000 kN, a loading rate of 6–10 mm/min, a displacement measurement accuracy of ±0.5 μm, and force measured by a high-precision load cell with an accuracy of ±0.02% F.S.

The puncture tests were likewise carried out using a commercial hydraulic testing machine with a self-made puncture test frame, equipped with a dedicated spherical puncture head (spherical radius 1200 mm, maximum projection diameter 1000 mm) with a 50 mm chamfer at the edge of the head to avoid the local sharp edges cutting the members; the maximum loading capacity was 1500 kN, and the loading rate and accuracy were the same as those of the tension testing machine.

### 2.2. Uniaxial Tension Test

During the test, the prepared ring-net specimen was installed in the fixtures of the testing machine to ensure natural spreading. The specimen was fixed in a four-side slidable manner, and the top was uniformly tensioned in-plane through a load-sharing beam. After zeroing the instruments, loading was performed in displacement control mode at 10 mm/min until the specimen fractured, and the load and displacement data were recorded in real time by the built-in data acquisition system. The tension test process is shown in Figure 3.

The force–displacement relationships of the specimens of all specifications are shown in Figure 4. All curves present a clear two-stage relationship: first, large deformation in tension dominates, and the load increases extremely slowly, with the specimen gradually tightening; in the second stage, the force increases sharply with displacement in a curve-rise manner. The mean breaking forces and peak displacements of the specimens are listed in Table 2; the breaking force increases with increasing net-ring specification (i.e., the number of wound strands), while the peak displacements of nets of different specifications vary within a small range. The summarized results are shown in Table 2.

### 2.3. Puncture Test

During the test, the surrounding rings of the specimen were fixed in the test frame by fixtures with the edge rings slidable, and the puncture head was aligned with the exact center of the specimen. After zeroing, loading was performed in displacement control mode at 10 mm/min until the specimen fractured, and the load and displacement data were recorded in real time. The puncture test process is shown in Figure 5 and Figure 6.

The resulting force–displacement curves of the specimens of all specifications are shown in Figure 6. Under the puncture loading state, the curves are nearly identical to those of the tension tests, also exhibiting the two-stage characteristics of initial tensioning followed by a sharp increase. The mean breaking forces and peak displacements of the specimens are listed in Table 3; the breaking force increases with increasing specification, while the peak displacement varies within a small range. As the initial dimensions of the puncture specimens are easier to control, the curves of specimens of the same specification are closer to each other. The summarized results are shown in Table 3.

### 2.4. Summary of the Tests

Through the uniaxial tension and puncture tests, the force-displacement curves, breaking forces, and peak displacements of 30 specimens of five specifications were systematically obtained. The results show that the specification has a significant influence on the breaking force, while the peak displacements of specimens of different specifications under the same loading state are relatively close [26].

The specification (number of steel wires *n*) mainly governs the breaking force through the parallel load-sharing of the wires, whereas the peak displacement is controlled by the geometric configuration of the ring net rather than by *n*. At failure, all rings reach a similar “arc-to-straight-line” deformation state determined by the ring geometry and the identical specimen dimensions (1100 mm gauge in tension, 3.2 m × 3.2 m in puncture); hence, peak displacements remain close across specifications, while breaking forces differ with *n*.

Whether in the tension or puncture tests, the force–displacement curves exhibit the same shape, showing both the two-stage characteristics of initial tensioning followed by a sharp increase, which lays a solid foundation for establishing a unified net model.

## 3. Establishment of the Improved Two-Stage Computational Model

Observations of the full-scale impact tests of flexible rockfall barriers show that in the impact span, the net suffers out-of-plane rockfall impact, and its overall deformation and loading state are close to those of the puncture test; in the side spans, the net slides and stretches along the structural length driven by the impact-span net, and its loading state is close to that of the tension test. This is also the reason why tension and puncture tests of specimens of the same specification were carried out simultaneously [7,27].

The test results show that the net presents apparently similar two-stage deformation characteristics in both the tension and puncture processes. In the early stage of loading, the net mainly experiences gradual contact from the initially “loose and soft” state, and the displacement–force relationship basically follows a linear law with slow and extremely small load growth; when the elongation further increases, the rings undergo a state change “from an arc to a straight line”, and the displacement–force curve shows a sharp increase in force with displacement.

The ring net undertakes two main loading cases in flexible rockfall protection systems: the tension case reflects the force–displacement response of the net under in-plane tension, and the puncture case reflects the bearing capacity of the net under concentrated loading (rockfall impact). Although the loading modes differ, both are formed by winding multiple steel wires into rings and carrying load in parallel, so their mechanical mechanisms are consistent [15]. Accordingly, this chapter adopts a unified normalization method and modeling procedure for the two cases to establish a unified-format piecewise constitutive model, describing the two loading states by different material parameters.

### 3.1. Normalization of the Test Results

The ring net is fabricated by winding several high-strength steel wires into rings; the specifications R8–R19 indicate that the rings are wound from 8 to 19 steel wires, respectively. To eliminate the influence of the number of steel wires on the force dimension, make the test results of different specifications comparable, and facilitate the establishment of a unified mechanical model, the force–displacement curves obtained from the tension tests were first normalized: the force was divided by the number of wound steel wires *n*, and the displacement by the initial gauge length of the ring net [26]. The normalization method can be expressed by the following equation:

(1) $$\bar{F}\;=\;\frac{F}{n},\chi \;\;=\;\frac{\Delta }{l_{0}}$$

where $\bar{F}$ is the normalized breaking force per wire, and χ is the normalized displacement. *l*_0_ is the initial reference length along the direction of the measured deformation variable *Δ*: it takes the value of the clamp spacing of 1100 mm for the tension case and 1600 mm (half the clamp spacing) for the puncture case, respectively.

For the tension case, the specimen is loaded in-plane in tension, and the normalized displacement *χ*_t_ is taken as the ratio of the measured displacement to the initial gauge length. The normalized displacement is calculated as follows:

(2) $$\chi_{t}\;=\frac{\Delta_{t}}{l_{0}}=\;\Delta_{t}/1100$$

where *χ*_t_ is the normalized displacement and *Δ*_t_ is the measured displacements (mm) for the tension case.

For the puncture case, the specimen is 3.2 m × 3.2 m, fixed on four sides and loaded at the center; the penetration depth at the loading point is converted through a geometric relationship into a dimensionless relative elongation “from the loading end to the edge of the specimen”. Here, a value of half the clamp spacing, namely 1600 mm, is uniformly used. The normalized displacement for the puncture case is calculated as

(3) $$\chi_{p}=\sqrt{1+{\left(\Delta_{p}/l_{0}\right)}^{2}}-1=\sqrt{1+{\left(\Delta_{p}/1600\right)}^{2}}-1$$

where *χ*_p_ is the normalized displacement and *Δ*_p_ is the measured displacements of puncture cases (mm).

Owing to the concentrated load applied at the center of the four-side-fixed specimen, the measured total puncture force *F* is distributed along the net toward the four fixed edges. To obtain a physically meaningful per-edge (unilateral) force for normalization, *F* was converted into the equivalent unilateral tension *T* of each edge through the geometric relationship:

(4) $$sin\alpha \;\;=\;\;\Delta_{p}/\sqrt{l_{0}^{2}+{\Delta_{p}}^{2}}$$

(5) T = F/(4·sinα) 

where α is the inclination angle of the net element with respect to the horizontal and *l*_0_ = 1600 mm is the distance from the center to the fixed edge (as defined in Equation (1)). Thus, the normalized puncture force is taken as *T*/*n* (per steel wire) instead of *F*/*n*.

A total of 15 tension curves and 15 puncture curves (three specimens for each of the five specifications) were all normalized uniformly.

### 3.2. Analysis of the Normalized Results

The normalized curves for both cases exhibit the typical two-stage form of “initial low-force tensioning segment—hardening rapid-rise segment—post-peak fracture”, with good consistency among specimens of the same group and obvious self-similar characteristics. Although both cases show good intra-group consistency, the normalized tension curves exhibit slightly larger dispersion than the puncture curves (Figure 7). This is because, in the tension test, the ring net is first clamped by the fixtures and then tensioned in-plane; the initial state of the specimen (e.g., the degree of slackness and the uniformity of the ring arrangement) is difficult to control precisely and inevitably varies among specimens, which propagates into the normalized curves. In contrast, the puncture test specimens have a well-controlled initial geometry: the ring net is fixed on four sides and loaded at the center, so the initial dimensions are easier to keep consistent among specimens, yielding closer normalized curves (Figure 7b).

Figure 7 presents the normalized curves for the tension and puncture cases together with the unified constitutive curve of a single steel wire (formally derived in Section 3.3, Equations (6) and (7)). For all five specifications, the normalized data collapse onto a single master curve that agrees well with this unified curve, confirming that the influence of the wire number *n* on the force and displacement dimensions has been effectively eliminated and that the two loading cases share an essentially identical normalized constitutive behavior. This self-similarity provides the basis for establishing the unified piecewise constitutive model in Section 3.3.

### 3.3. Unified Piecewise Constitutive Model

Since the normalized curves for the two cases are consistent in shape and self-similar, a unified-format piecewise constitutive model can be established. The normalized per-wire relation $\bar{f}(\chi )$ is described by a “linear tensioning segment + power-hardening segment” up to the peak point [28]:

(6) $$\bar{f}\left(\chi \right)=\;k_{0}\chi ,0\leq \chi \leq \chi_{1}$$

(7) $$\bar{f}\left(\chi \right)=F_{1}+A{\left(\chi -\chi_{1}\right)}^{p},\chi_{1}\leq \chi \leq \chi_{max}$$

where *k*_0_ is the stiffness of the tensioning segment; *χ*_1_ is the normalized displacement at the end of the tensioning segment, and *F*_1_ = *k*_0_·*χ*_1_ is the corresponding normalized force; *p* is the power-hardening exponent; *A* = (*F*_max_ − *F*_1_)/(*χ*_max_ − *χ*_1_) is the hardening coefficient; *χ*_max_ and *F*_max_ are the normalized peak displacement and peak force, respectively. The piecewise form of Equation (6) itself guarantees continuity of the model, and the parameters *p* and *A* are determined by fitting Equation (7) to the measurement data using the least-squares method.

Because the steel wires share the load in parallel, the overall force–displacement relation of the ring net is obtained by multiplying the single-wire law $\bar{f}(\chi )$ by the number of wires *n*:

(8) $$F\left(\chi ;n\right)=\;n\bar{f}(\chi )$$

The parallel-superposition mechanism is further confirmed quantitatively by the linear dependence of the total peak bearing capacity on *n*. Fitting the total peak bearing capacity of each specification against the number of steel wires *n* gives, for the tension and puncture cases, respectively [29]:

*F*_t,max_ = *a*_t_·*n* + *b*_t_ (9)

*F*_p,max_ = *a*_p_·*n* + *b*_p_ (10)

where *F*_t,max_ and *F*_p,max_ are the total peak bearing capacities in the tension and puncture cases, respectively; *n* is the number of steel wires wound in one ring; and *a*_t_, *b*_t_, *a*_p_, *b*_p_ are the regression coefficients obtained by linearly fitting the measured peak bearing capacity of each specification (*n* = 8, 9, 12, 16, 19) against *n*.

It should be noted that *F*_t,max_ and *F*_p,max_ are the total peak capacities consistent with the normalized curves: for the tension case they are the total tension capacities, whereas for the puncture case, they are the unilateral (per-edge) peak capacities *T* converted from the total puncture forces *F* through Equations (4) and (5).

The total bearing capacities of both cases are strongly linearly correlated with *n*, with power-law exponents of about 1.04 and 1.18, respectively, both close to or slightly larger than 1, indicating that the ring net follows the “parallel superposition” bearing mechanism in both tension and puncture: each additional steel wire increases the total tension bearing capacity by about 35 kN and the unilateral oblique puncture bearing capacity by about 39 kN. After normalization, the peak bearing force per steel wire stays essentially constant across all specifications—about 32.5 kN for tension and 31.3 kN for the equivalent unilateral puncture value—regardless of the number of wires wound into the ring (*n* = 8–19), indicating that each wire shares a nearly equal failure load and the total capacity scales linearly with *n* [26].

The model parameters were calibrated by least-squares fitting of the normalized curves to Equations (6) and (7). For the tension case, the 15 normalized tension curves were fitted separately; the coefficient of determination *R*^2^ of each specimen was not lower than 0.97, with a mean of 0.98 [12]. Taking the mean parameter values, the unified parameters for the tension case are *k*_0t_ = 8.75 kN, *p*_t_ = 6.51, *χ*_1t_ = 0.097, *χ*_t,max_ = 0.313, *F*_t,max_ = 32.54 kN, *F*_1t_ = 0.852 kN, and *A*_t_ = 6.93 × 10^5^. For the puncture case, the 15 normalized puncture curves were fitted separately, with a mean *R*^2^ of 0.983 [11]; the unified parameters are *k*_0p_ = 42.4 kN, *p*_p_ = 5.78, *χ*_1p_ = 0.026, *χ*_p,max_ = 0.227, *F*_p,max_ = 33.4 kN, *F*_1p_ = 1.11 kN, and *A*_p_ = 3.44 × 10^5^. Accordingly, the tension and puncture cases adopt the same constitutive form and differ only in the parameters, as compared in Table 4.

It can be seen from Table 4 that the unilateral oblique peak force of the puncture case is comparable to that of the tension case (about 32–33 kN), but the tensioning stiffness of the puncture case is about 4.8 times that of the tension case, and the power-hardening exponent and peak displacement are smaller, reflecting the mechanical characteristics of more concentrated loading and faster deformation of the net under concentrated loading.

### 3.4. Model Verification

On the basis of the unified piecewise constitutive model and its calibrated parameters in Section 3.3, the overall force–displacement relations of all five specifications are predicted from Equation (8) and compared with the measurements; the results are presented in Figure 8 and Figure 9 for the tension and puncture cases, respectively. In both figures, the dashed lines denote the measured data of each specification and the black solid lines denote the theoretical predictions obtained from the two-stage model established above.

For all five specifications, the predicted curves pass through the center of the normalized measured data and agree well with the measurements in the pre-peak stage: the predicted total force–displacement curves are consistent with the measured ones in variation trend and agree well up to the peak. The relative error between the predicted and measured peak bearing forces is about 8.2% on average for the tension case and 7.8% for the puncture case (see Figure 8f and Figure 9f), and the coefficient of determination between the predicted and measured peak bearing capacities is 0.94 for the tension case and 0.976 for the puncture case. Beyond the peak, the model and the measurements naturally diverge as fracture of the steel wires initiates, which lies outside the scope of the present two-stage model. These results verify the rationality and applicability of the unified model for both loading cases.

### 3.5. Summary of This Chapter

This chapter established a unified-format mechanical model for the tension and puncture cases of the ring net. The test data of the two cases were first normalized (force divided by the number of steel wires *n*, displacement non-dimensionalized according to the in-plane elongation and the geometric oblique length relationship, respectively), revealing that both cases follow the “parallel superposition” bearing mechanism; a unified piecewise constitutive model $\bar{f}$(χ) was then established, describing the two loading states by the five parameters (*k*_0_, *p*, *χ*_1_, *χ*_max_, *F*_max_), and the force–displacement relationship for any specification with *n* steel wires is given by F(χ; *n*) = *n*·$\bar{f}$(χ). The unified model is concise in form, clear in physical meaning, and of high fitting accuracy (*R*^2^ ≥ 0.98), with the differences between the puncture and tension cases quantitatively reflected by the parameters, providing a unified theoretical basis for prediction of the mechanical properties and the engineering design of ring nets.

## 4. Verification of the Model

To verify the accuracy of the established mechanical model of the metal ring net, a full-scale impact test of the overall structure with 1500 kJ energy was carried out [3,30,31]. A comparative numerical simulation model was then established using the ring-net model established above, and the accuracy of the model was verified from several aspects, including the overall structural deformation, the internal forces of the main members, and the activation states of the structural energy dissipators.

### 4.1. Overview of the Full-Scale Impact Test

The test flexible rockfall barrier was designed and installed according to the actual passive flexible protection system; the net body was suspended on the test wall and fixed to the anchor points by a cable system composed of support ropes and pressure-relief rings, and the impact energy was absorbed and intercepted jointly through the overall structural deformation, the tensioning of the support ropes, and the energy dissipation of the pressure-relief rings. The basic configuration parameters of the test flexible rockfall barrier are listed in Table 5.

A large-mass drop hammer was released in free fall along the vertical direction to impact the middle of the net face, with an impact energy of 1500 kJ and an impact velocity near the net of not less than 25 m/s. Force sensors were arranged near the anchor ends of the steel wire ropes to record the cable force time histories, and a high-speed camera was used to record the impact process of the net face. The full-scale impact test is shown in Figure 10.

### 4.2. Establishment of the Numerical Simulation Model

Based on the piecewise constitutive mechanical model of the metal ring net established in Section 3, an overall structural numerical simulation model corresponding to the full-scale impact test was established using the explicit finite element software LS-DYNA (version R11.0, MPP double-precision solver). The model is composed of the ring net (equivalent truss elements), the support ropes and anchor ropes (cable elements), the pressure-relief rings (energy-dissipating elements) and the supporting steel structure, as shown in Figure 11. The modeling procedure, the element types and the material parameters of the main members are described in detail below.

The ring net adopted in the numerical model is the R12-3.0-300 specification: each ring has a nominal ring diameter *D* = 300 mm (initial ring radius *R* = 150 mm) and is wound from *N* = 12 high-strength steel wires with a wire diameter *d*_0_ = 3.0 mm (nominal strength 1770 MPa). The steel wire itself has a Young’s modulus *E*_s_ = 2.06 × 10^5^ MPa, a Poisson’s ratio *ν* = 0.3 and a density of *ρ* = 7850 kg/m^3^. It should be noted that the equivalent ring element is not directly assigned the steel-wire modulus; its equivalent elastic modulus is derived from the piecewise constitutive model, as described below.

Following the discretization scheme of Ref. [27], nodes were established at the inter-ring contact positions and at the centers of the rings, and four circular-section truss elements were defined by connecting these nodes, so as to complete the equivalent discretization of the whole ring net in a head-to-tail manner. To preserve the inertial effects, the mass equality was maintained by equalizing the volumes of the equivalent element and the original ring; since the initial length of each equivalent element equals the initial ring radius *R* and the material density remains unchanged, the section radius of the equivalent truss element is obtained as

(11) $$r_{truss}=r\sqrt{n\pi /2}$$

where *r* is the radius of a single steel wire (*r* = 1.5 mm) and *n* is the number of wound wires (*n* = 12). Substituting the parameters of the R12-3.0-300 ring gives the section radius of the equivalent element *r*_truss_ = 28.27 mm, and the corresponding equivalent section area *A*_eq_ = 2511.5 mm^2^.

To be compatible with the standard finite element formulation, the force–displacement (P–δ) restoring-force relationship of the ring was converted into the material stress–strain relationship of the equivalent bar element, with the equivalent strain and equivalent stress given by

(12) $$\varepsilon =\frac{\delta }{R}$$

(13) $$\sigma =\frac{P}{\pi r_{truss}^{2}}$$

where *δ* is the deformation of the ring and *P* is the tension of the ring. Accordingly, the equivalent computational model of the R12-3.0-300 ring net is fully defined by the equivalent geometry parameters (*r*_truss_ = 28.27 mm, *A*_eq_ = 2511.5 mm^2^, initial element length *R* = 150 mm) together with the material parameters listed in Table 6.

The material of the equivalent ring element was assigned the piecewise constitutive model established in Section 3, i.e., substituting Equations (11)–(13) converts this normalized two-stage law into the equivalent stress–strain (σ–ε) relationship assigned to the truss elements. Because the equivalent element possesses a much larger section area than the sum of the steel wires, the steel-wire modulus (*E*_s_ = 2.06 × 10^5^ MPa) cannot be used directly; otherwise, the stiffness would be overestimated, and the deformation of the net underestimated. Moreover, since the equivalent stress–strain law is two-stage, the equivalent elastic modulus of the ring element is not a single constant: only the linear tensioning segment has a fixed initial tangent modulus *E*_t0_ = dσ/dε, which, for the tension case, is evaluated as $E_{t0}\;\;$≈ 5.70 MPa (fixture spacing *L*_t_ = 1100 mm). For the puncture case, the tensioning segment is approximately linear, and the secant modulus at the end of the tensioning segment is taken as *E*_t0_ ≈ 2.15 MPa, obtained through the geometric relation. In the power-hardening segment, however, the tangent modulus grows progressively with strain and is given by the derivative of the σ–ε relationship; hence, the overall equivalent response is genuinely two-stage rather than described by a single fixed modulus.

Since the ring nets in different spans of the overall structure are subjected to different loading states, the structure was divided by spans in modeling: the ring nets in the two side spans adopt the piecewise constitutive model calibrated for the tension case, whereas the ring nets in the middle (impact) span adopt the piecewise constitutive model calibrated for the puncture case. The normalized piecewise law of each case was restored to the actual load–displacement relationship according to the geometric and material parameters of the rings, converted into the equivalent stress–strain relationship, and assigned to the corresponding truss elements.

The remaining structural members were modeled in accordance with the actual full-scale test configuration, with the model adopting the same horizontal installation as the impact test to facilitate comparison with the literature and experimental results. The support ropes and anchor ropes were modeled using cable elements that can transmit only uniaxial tension, so as to reflect the tensioning process of the steel-wire ropes; the supporting steel columns were modeled using user-defined beam elements. The material parameters of these members are listed in Table 7.

The anchoring ends of the support ropes and anchor ropes were fully constrained in all directions. The column feet of the supporting steel columns were constrained against translation in the three directions while releasing the rotation about the vertical axis, so as to simulate the actual installation in which the column feet can rotate vertically; in addition, spring elements were arranged at the column feet to simulate the finite horizontal sway of the column feet. The slidable connection between the ring net and the support ropes, i.e., the way the ring net is threaded onto the support ropes through the shackles, was simulated by a slidable point-to-line connection; the way the upper and lower support ropes pass through the sliding bearings at the column head and column foot was simulated by seat-belt (belt) element pairs.

The sliding contact between the ring net and the support ropes and the relative sliding between the support ropes and the anchor points were simulated by sliding contact interfaces. A Coulomb friction model was adopted, and the sliding friction coefficient *μ* was set to 0.1. The contact algorithm, the contact type and the friction coefficient are summarized in Table 8.

The impact condition was consistent with the test: the impact block was released in free fall along the vertical direction to impact the center of the middle span, applying an impact energy of 1500 kJ to the structure, and the nonlinear dynamic response of the structure was solved by explicit time integration with the LS-DYNA R11.0 MPP double-precision solver. The mesh statistics are listed in Table 9.

### 4.3. Verification Results and Analysis

To verify the accuracy of the net mechanical model, the numerical results were compared with the full-scale test measurements from three aspects: the support-rope force time histories, the activation states of the pressure-relief rings, and the overall structural impact interception deformation [24,32,33].

As the cable force time histories finally obtained in the full-scale impact test were limited, this section only compares the force time histories of the upper and lower support ropes and the two inner upper anchor ropes of the impact span obtained from the test and the calculation. The results show that the calculated variation trend of the support-rope force with time is consistent with the measured curves, and the time and magnitude of the peak force agree well, with good overall consistency between the two, verifying that the ring-net mechanical model can accurately reflect the process of the impact load transmitted in the net and the resulting loading on the support ropes. The comparison of the cable force time histories is shown in Figure 12.

In the test, the activation characteristic of the pressure-relief rings is manifested as an obvious reduction of the ring diameter and straightening of the round tube [34]. As shown in Table 10, the ranking of the energy dissipation of the pressure-relief rings of each member is consistent between the test and the numerical simulation, namely the lower support-rope pressure-relief rings (about 400 kJ) > the upper anchor-rope pressure-relief rings (about 375 kJ) > the upper support-rope pressure-relief rings (about 220 kJ), indicating that the model can correctly reflect the distribution of the impact energy among the energy-dissipating members. The average relative error of the energy dissipation of the three pressure-relief rings is about 5.2%, among which the errors of the upper anchor rope (1.7%) and the lower support rope (3.8%) are small and in good agreement; the error of the upper support-rope pressure-relief ring is the largest (about −10.0%), with the calculated value being low, which may be related to the simulation differences in the load-transfer path of the upper support rope and the activation sequence of the pressure-relief rings, and is still within the acceptable engineering error range. The activation states of the upper and lower support-rope pressure-relief rings and the comparison of the energy dissipation are shown in Figure 13 and Figure 14.

Comparing the maximum impact interception deformation of the protection net after impact (the maximum vertical displacement of the net face), the measured maximum interception deformation in the full-scale test was 8.8 m, and the calculated result of the numerical simulation was 8.23 m, with a relative error of about 6.5%, showing good agreement. The consistency of the maximum interception deformation indicates that the ring-net mechanical model can well reflect the force and deformation state of the overall structure under impact [32,35].

### 4.4. Summary of This Chapter

Through the comparison and verification of the 1500 kJ full-scale impact test and the LS-DYNA numerical simulation results based on the ring-net mechanical model, good consistency was achieved in the three aspects of the upper and lower support-rope force time histories, the activation states of the pressure-relief rings, and the maximum impact interception deformation of the overall structure. The verification results show that the established piecewise mechanical model of the metal ring net can well reflect the force and deformation state of the overall structure, accurately describe the transmission of the impact force in the ring net and the activation process of the energy dissipators (pressure-relief rings) in the actual structure, and can be used for the overall design and analysis of passive flexible protection systems.

## 5. Conclusions

Through systematic uniaxial tension and puncture tests, normalization analysis and modeling, as well as full-scale impact test and numerical simulation verification, this paper established a two-stage computational model that can more realistically reflect the mechanical behavior of the metal ring net and its synergistic working mechanism with the energy dissipators. The following main conclusions are drawn.

(1) The uniaxial tension and puncture tests of 30 ring-net specimens of five specifications from R8 to R19 show that the force-displacement curves of the net under both loading cases present the two-stage deformation characteristics of “initial tensioning—sharp increase”, the breaking force increases approximately linearly with the number of steel wires *n* following the “parallel superposition” bearing mechanism, and the peak displacement remains relatively stable.
(2) After the normalization of the test data (force divided by the number of steel wires *n*, displacement non-dimensionalized according to the in-plane elongation and the geometric oblique length relationship, respectively), the normalized curves for nets of different specifications under the two loading cases both exhibit obvious self-similar characteristics. On this basis, a unified-format piecewise constitutive mechanical model was established, using a “linear tensioning segment + power-hardening segment” to describe the whole process of the net loading up to the peak point, with the tension and puncture cases adopting the same constitutive form and distinguished only by different parameters. The coefficient of determination *R*^2^ of each specimen was not lower than 0.97 (with mean values of 0.98 for tension and 0.983 for puncture), and the model can accurately describe the force and deformation characteristics of ring nets of different specifications.
(3) Based on the established net mechanical model, an overall structural numerical simulation model corresponding to the 1500 kJ full-scale impact test was established using LS-DYNA, in which the two side spans adopt the tension-case constitutive and the middle impact span adopts the puncture-case constitutive. Compared with the full-scale test results, good consistency was achieved in the upper and lower support-rope force time histories, the activation states of the pressure-relief rings, the maximum impact interception deformation of the overall structure (measured 8.8 m, calculated 8.23 m, relative error about 6.5%), and the energy-dissipation distribution of the pressure-relief rings (average relative error about 5.2%), verifying the accuracy and applicability of the net mechanical model.
(4) The verification results show that the established two-stage computational model can accurately reflect the force and deformation responses of the ring net under in-plane tension and out-of-plane concentrated impact in the actual structure, correctly describe the transmission of the impact force in the net and the activation and energy dissipation process of the energy dissipators (pressure-relief rings), and can be used for the refined design, energy-distribution assessment, and safety analysis of flexible rockfall protection systems.

## Figures and Tables

**Figure 1:**
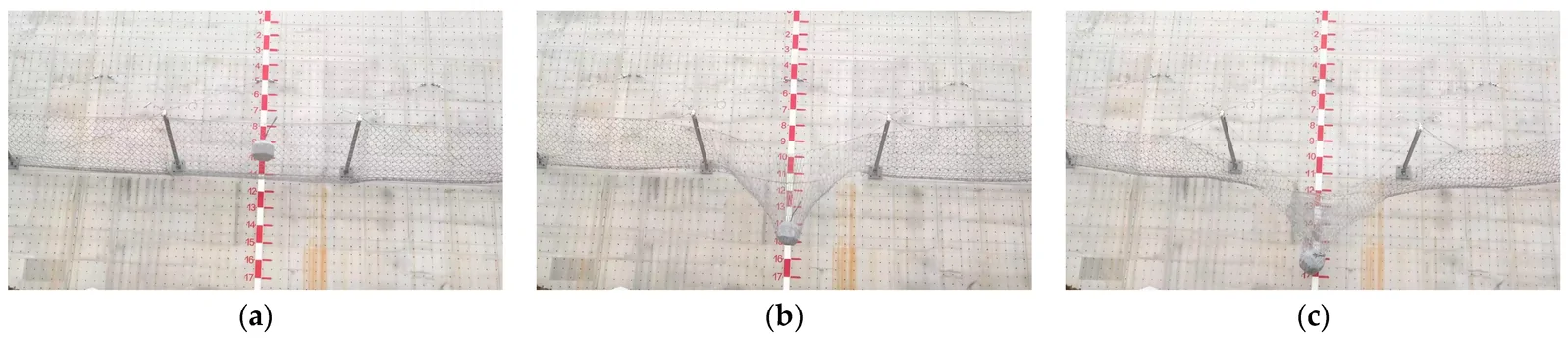
Schematic of structural impact interception. (**a**) Contact net surface. (**b**) Net panel deformation. (**c**) Support-rope energy-dissipator deformation.

**Figure 2:**
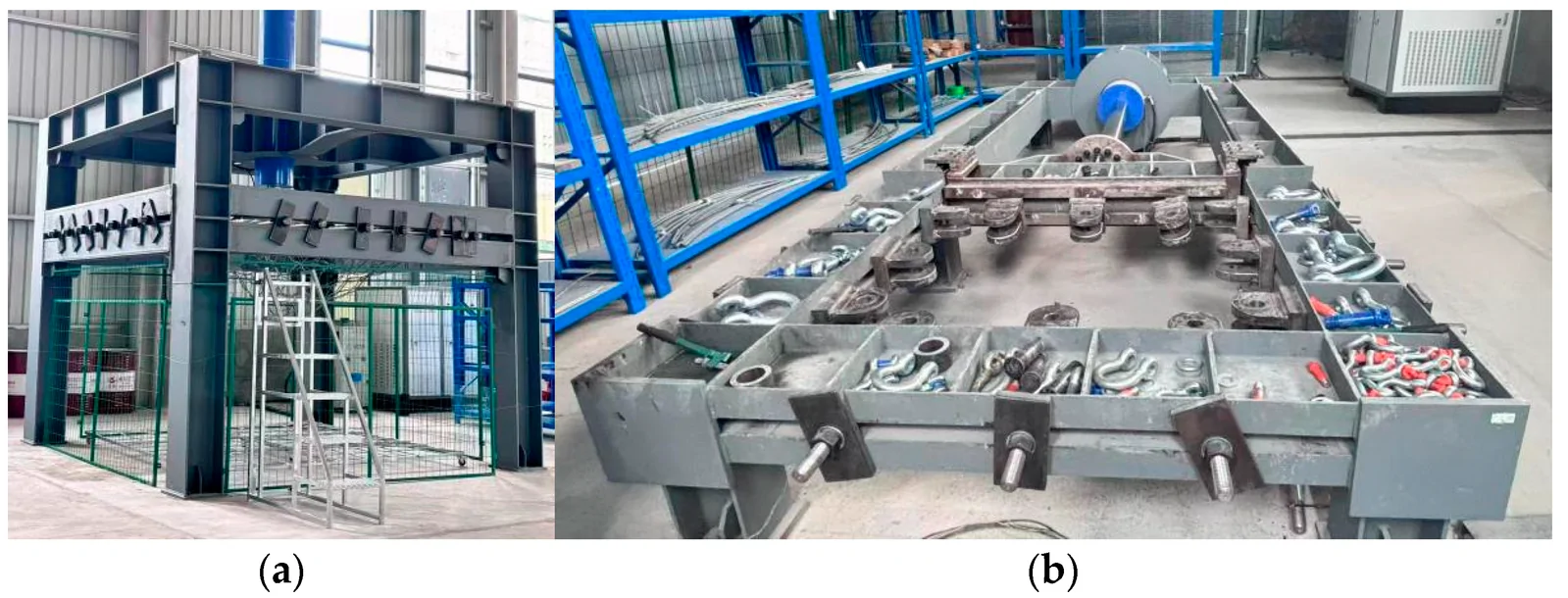
Test instruments. (**a**) Puncture testing machine. (**b**) Tension testing machine.

**Figure 3:**
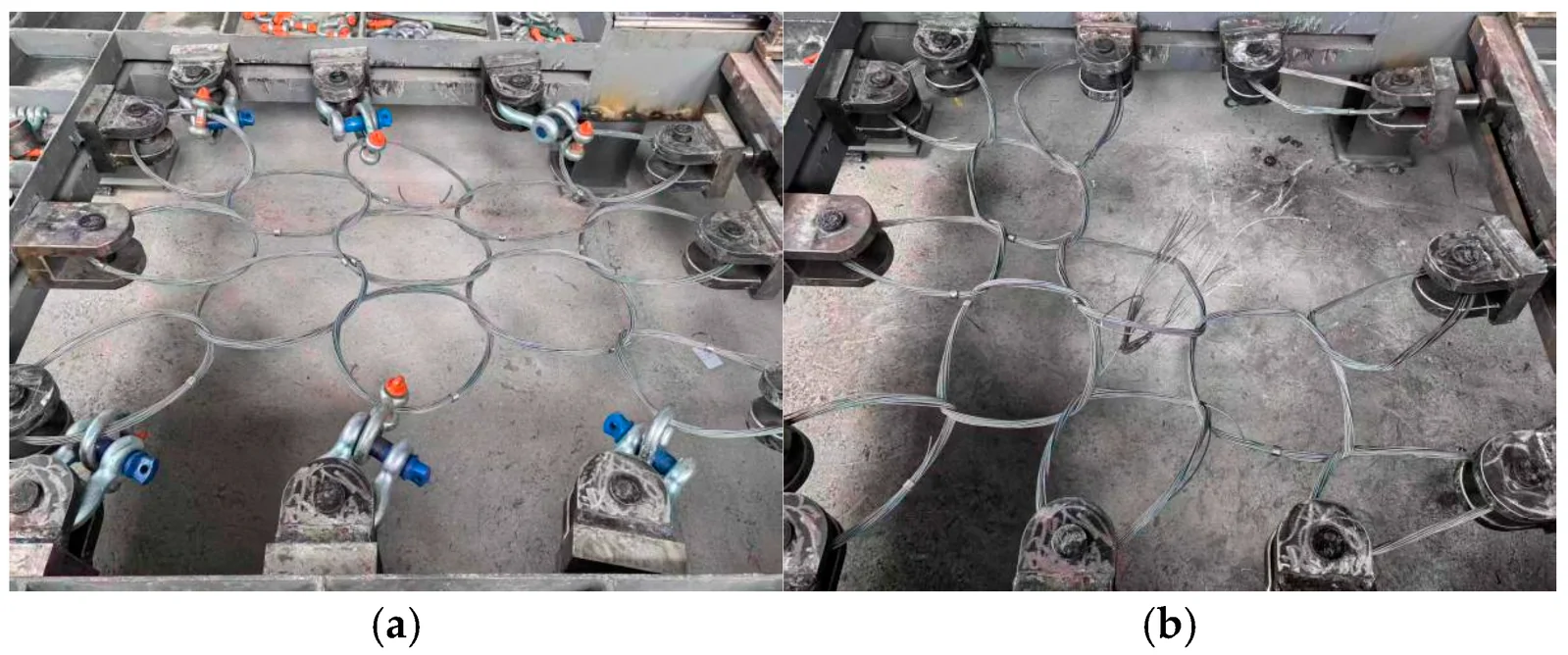
Tension test. (**a**) Initial tensioning. (**b**) Failure state.

**Figure 4:**
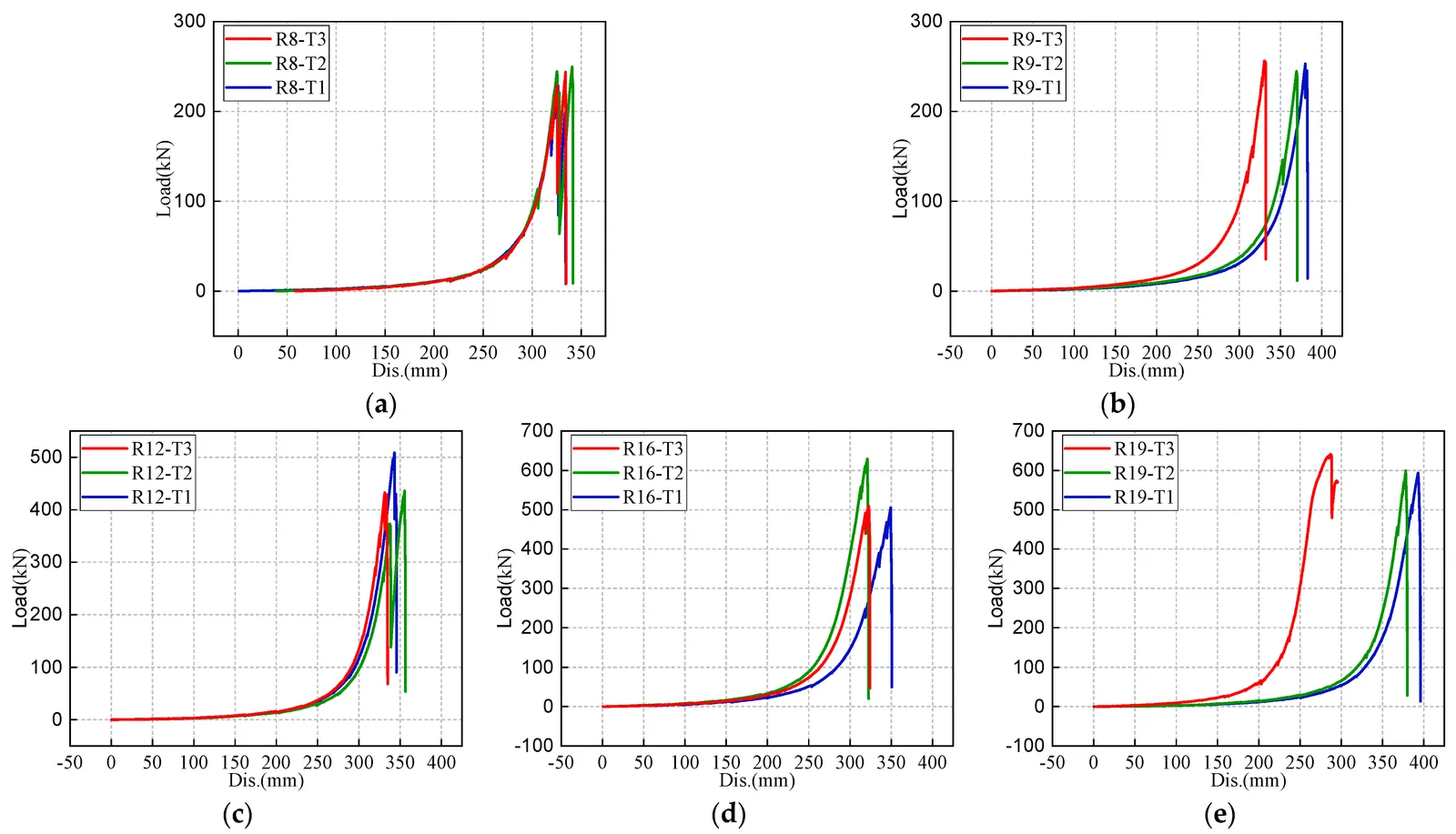
Tension test results. The notation “Rx-Tk” denotes the k-th repeated specimen of specification Rx (e.g., “R8-T1” is the first specimen of the R8-0.3-300 ring net); the other specifications follow the same convention. (**a**) R8-3.0-300, (**b**) R9-3.0-300, (**c**) R12-3.0-300, (**d**) R16-3.0-300, (**e**) R19-3.0-300.

**Figure 5:**
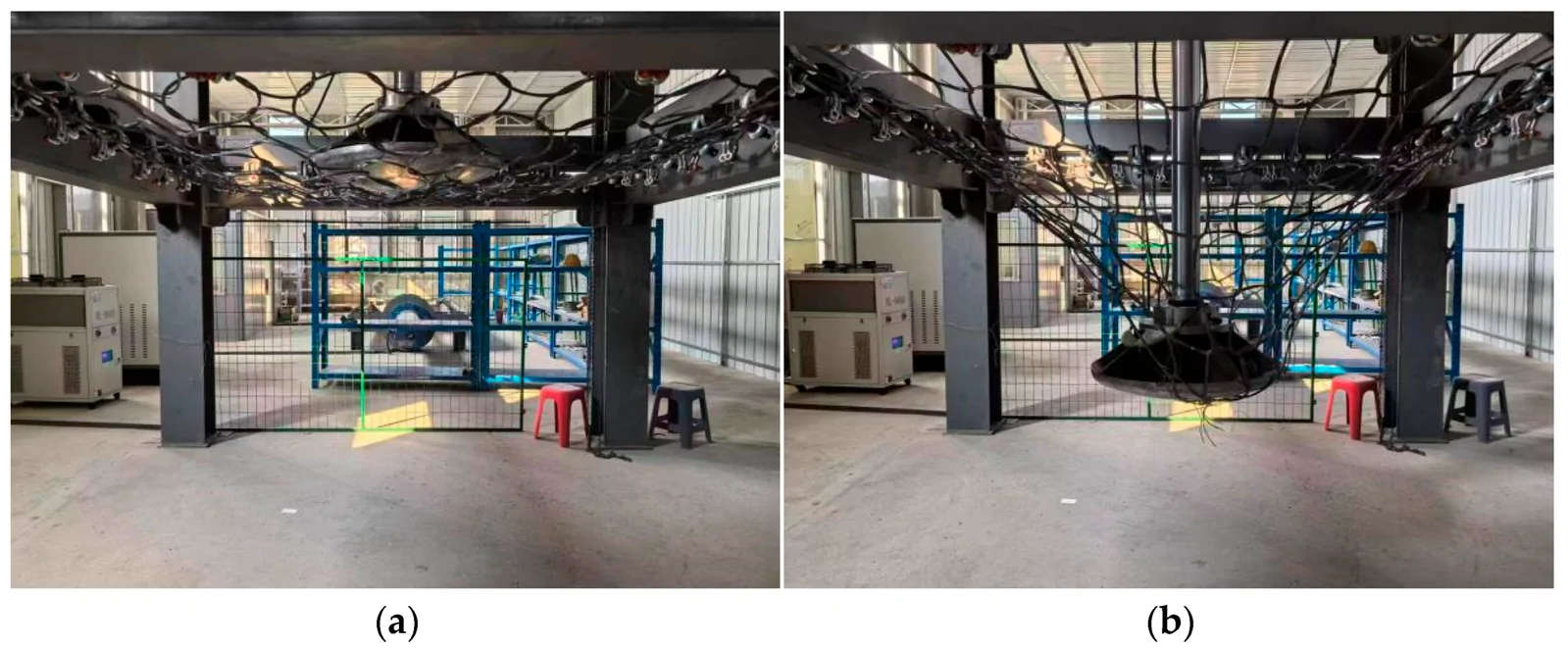
Puncture test. (**a**) Initial tensioning. (**b**) Failure state.

**Figure 6:**
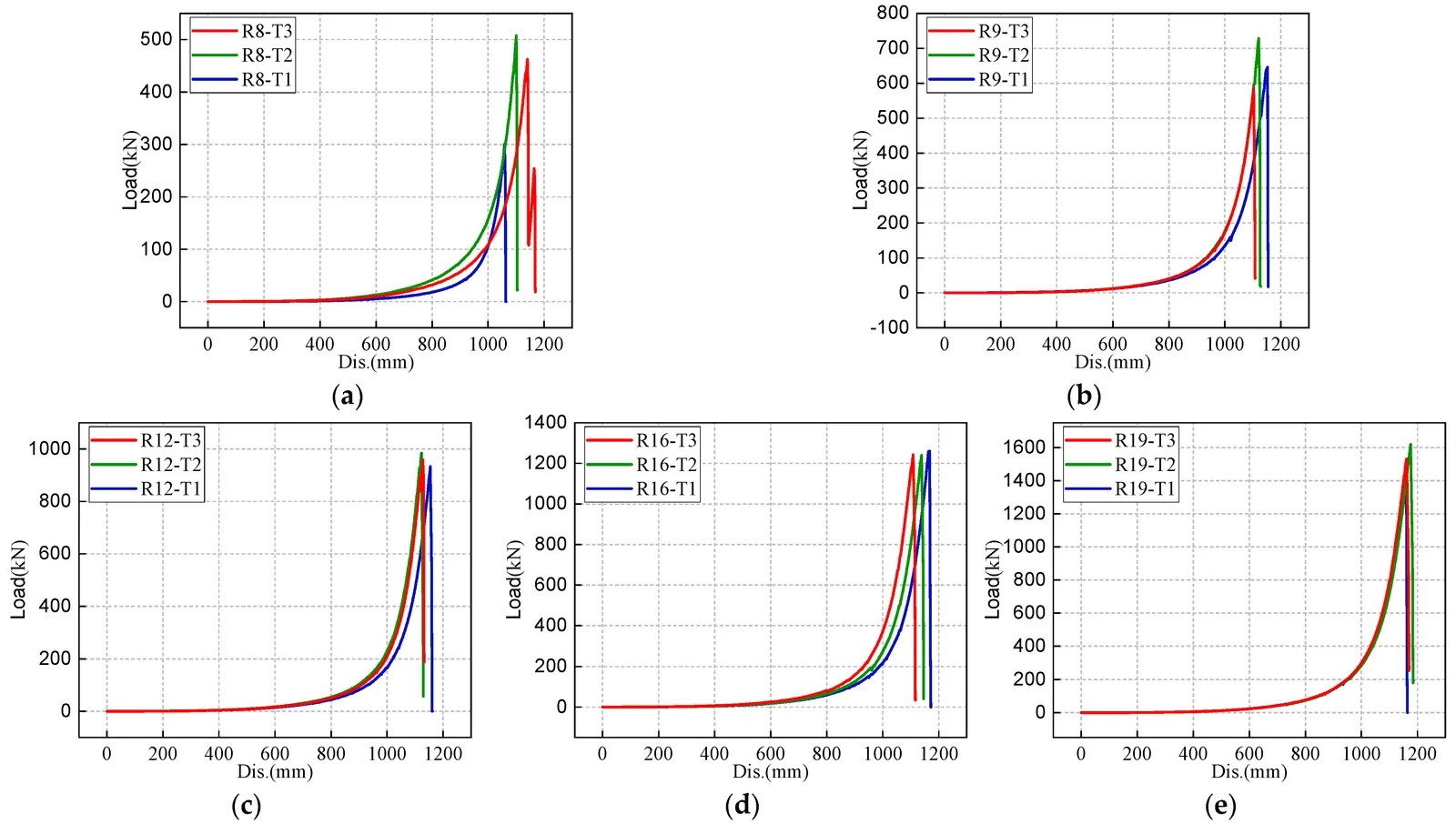
Puncture test results. (**a**) R8-3.0-300, (**b**) R9-3.0-300, (**c**) R12-3.0-300, (**d**) R16-3.0-300, (**e**) R19-3.0-300.

**Figure 7:**
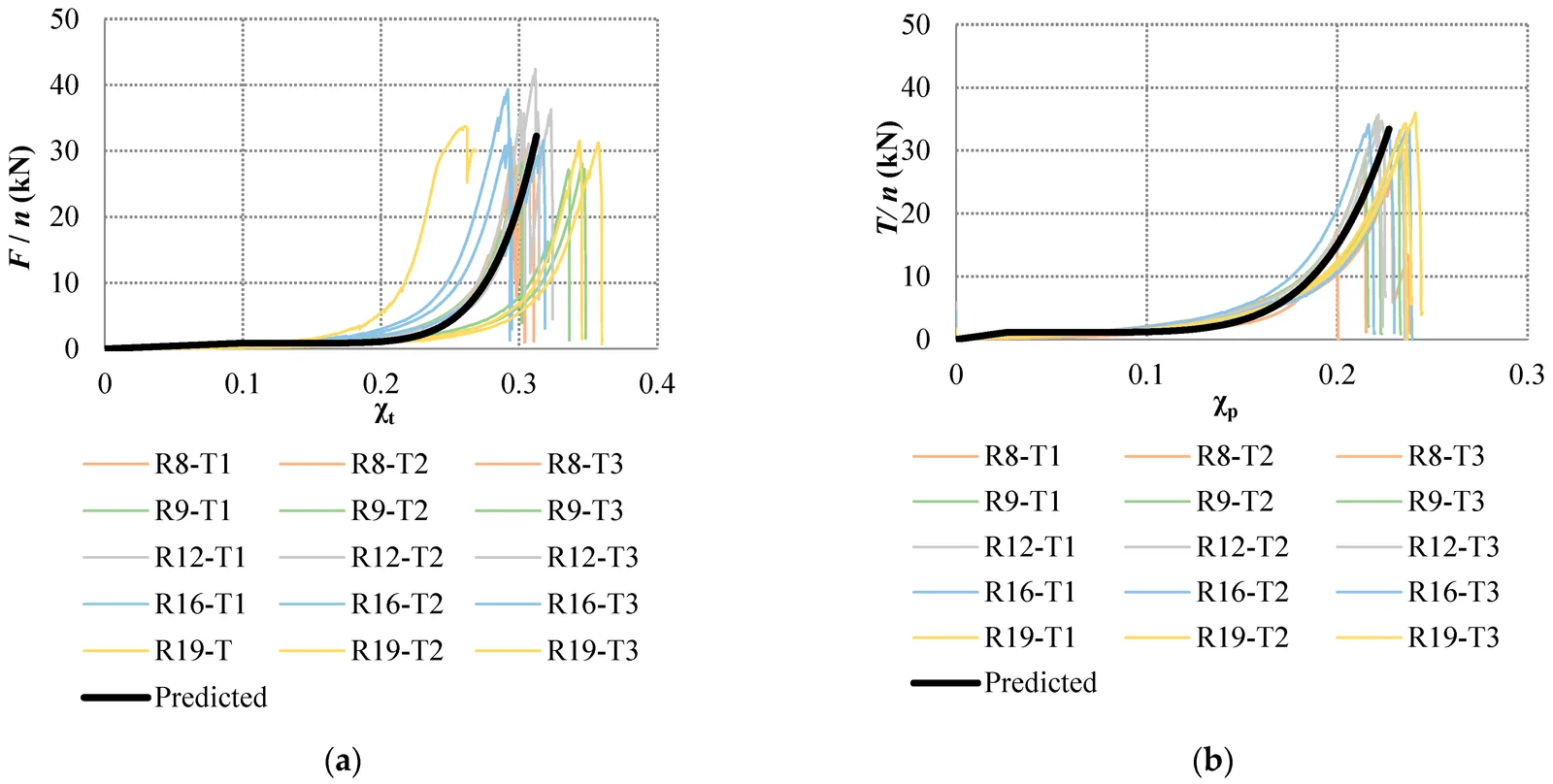
Normalization of the test results and comparison with the predicted single-wire curve. (**a**) Tension test. (**b**) Puncture test.

**Figure 8:**
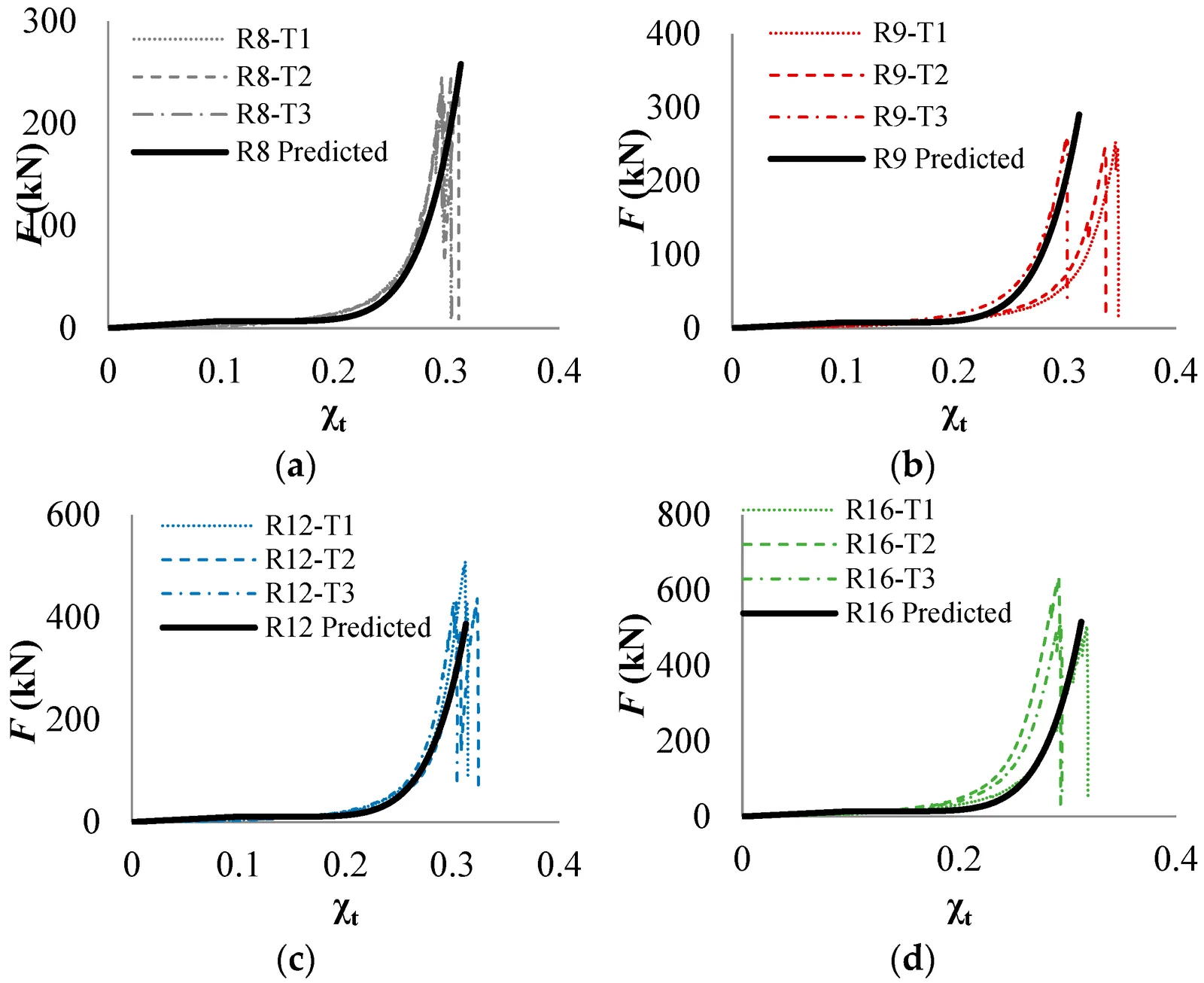

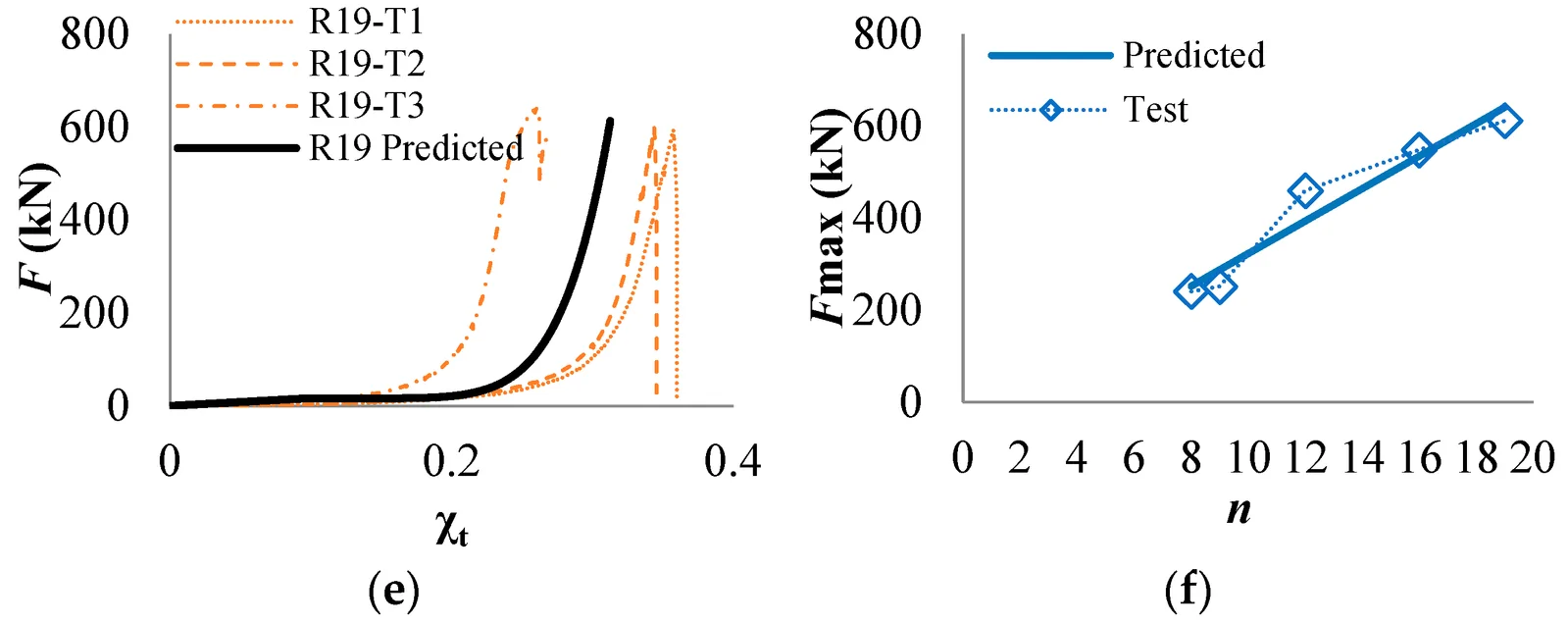
Comparison between the predicted curves of the tension parallel-superposition model and the measurements of each specification. (**a**) R8, (**b**) R9, (**c**) R12, (**d**) R16, (**e**) R19, (**f**) Comparison of breaking forces.

**Figure 9:**
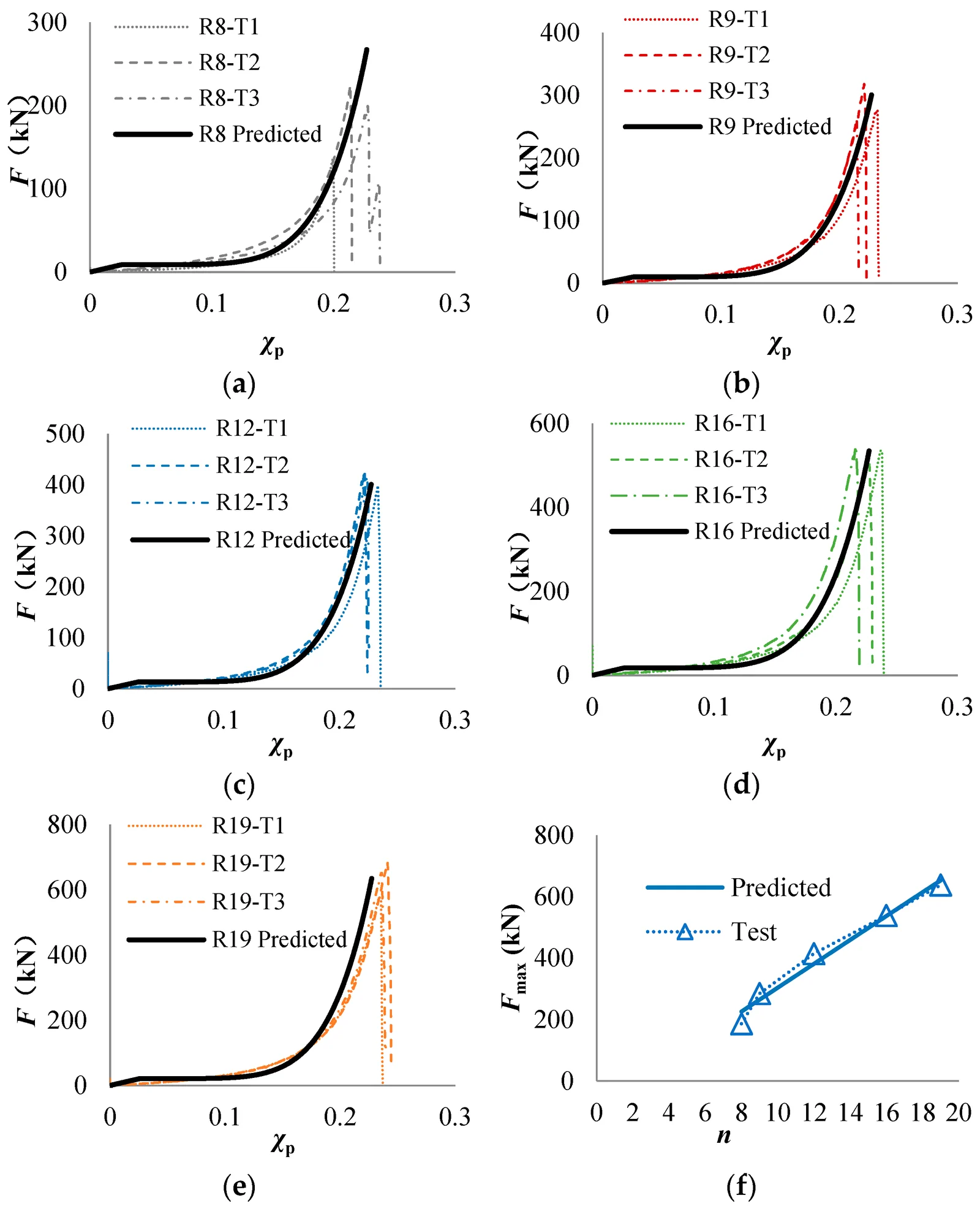
Comparison between the predicted curves of the puncture parallel-superposition model and the measurements of each specification. (**a**) R8, (**b**) R9, (**c**) R12, (**d**) R16, (**e**) R19, (**f**) Comparison of breaking forces.

**Figure 10:**
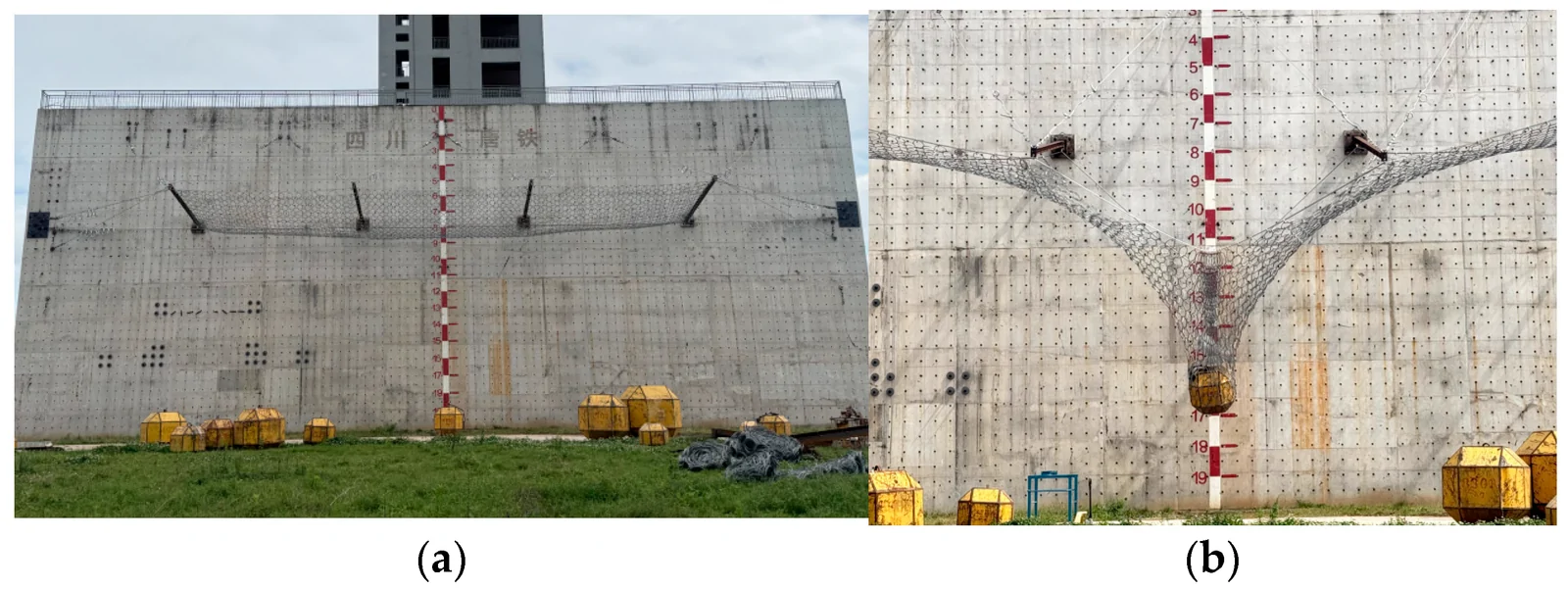
Full-scale impact test. (**a**) Before the test. (**b**) Impact interception state.

**Figure 11:**
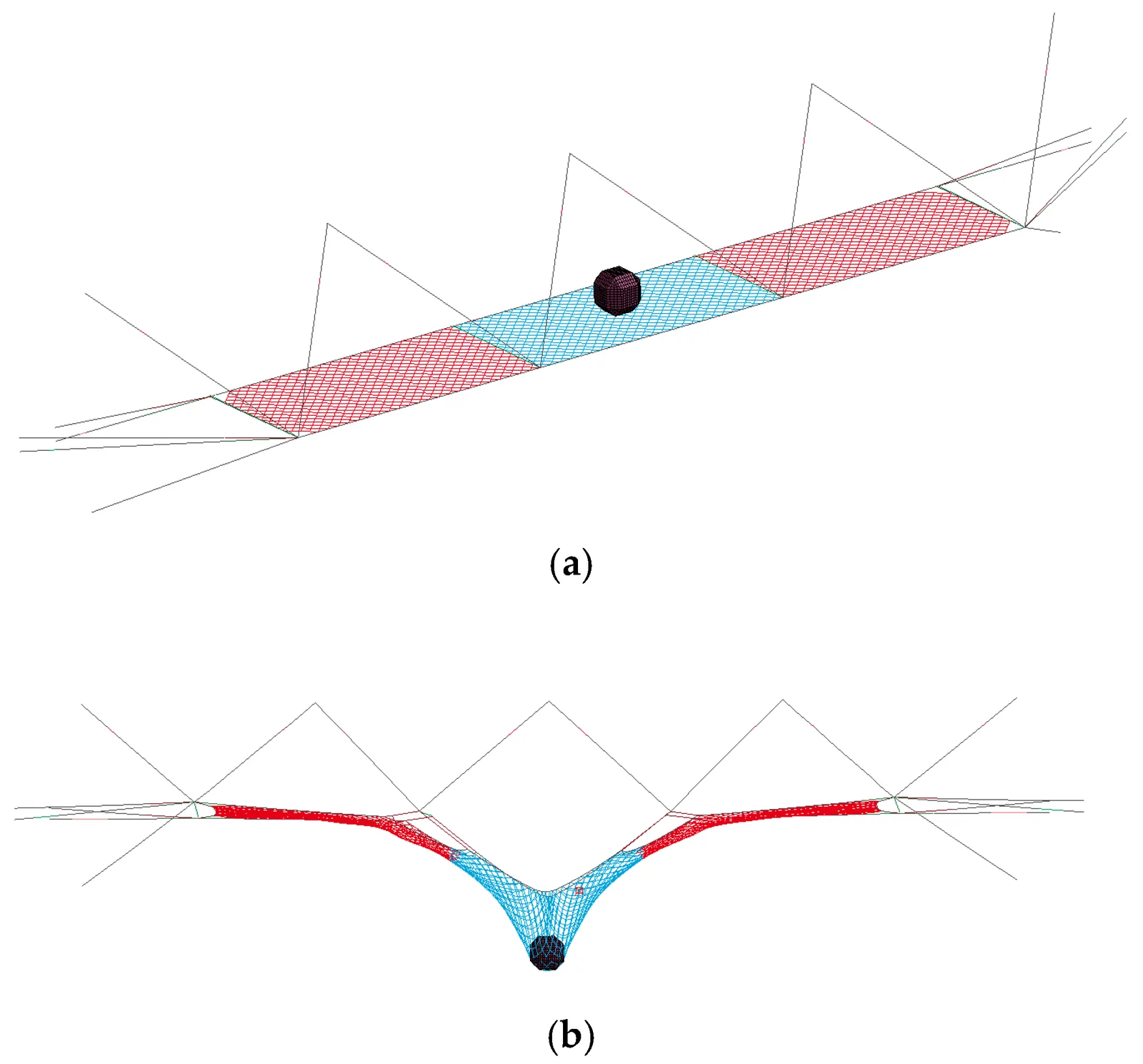
Numerical simulation calculation. (**a**) Numerical simulation model. (**b**) Final calculation results.

**Figure 12:**
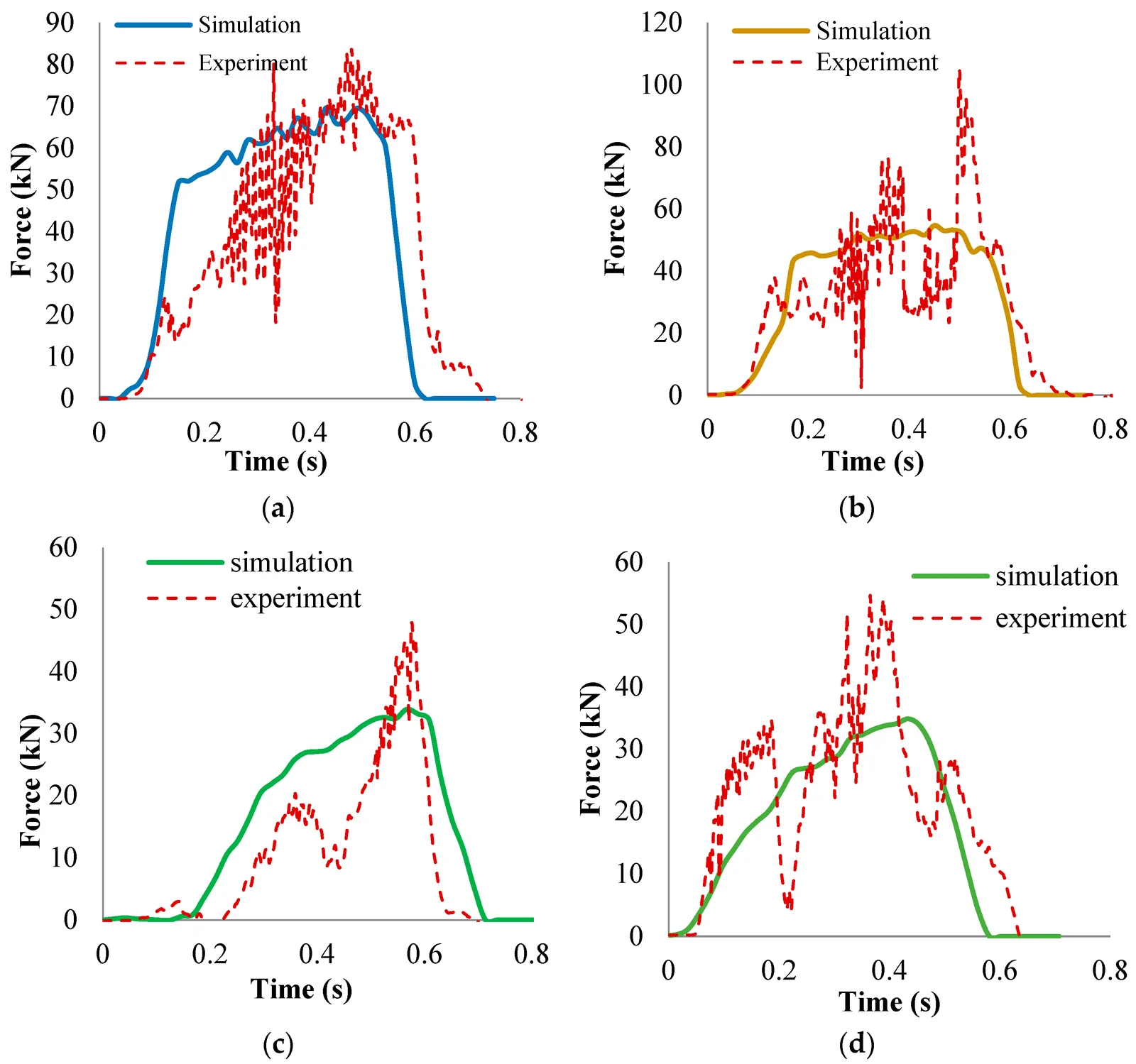
Comparison of the cable force time histories. (**a**) Lower support rope. (**b**) Upper support rope. (**c**) Inner upper anchor rope at the left of the impact span. (**d**) Inner upper anchor rope at the right of the impact span.

**Figure 13:**
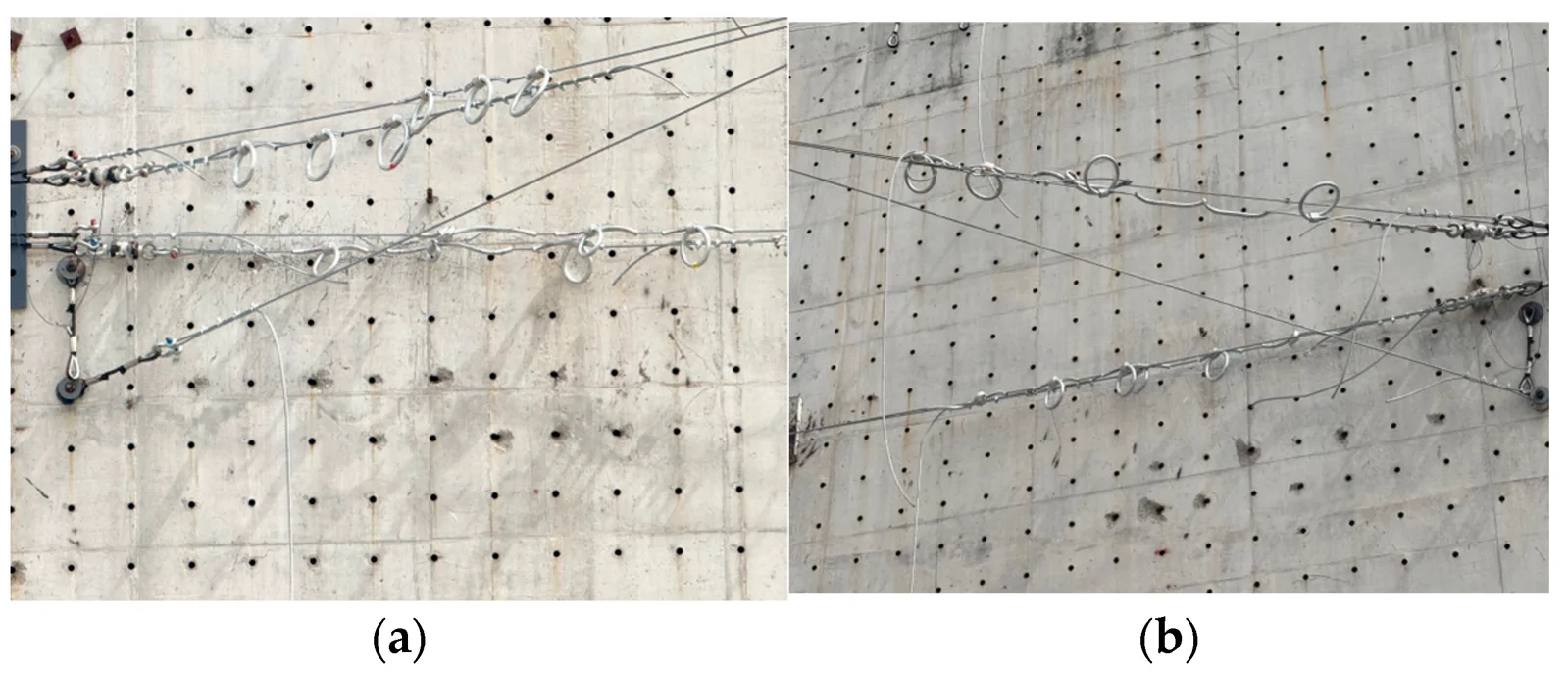
Activation states of the upper and lower support-rope pressure-relief rings. (**a**) Left side. (**b**) Right side.

**Figure 14:**
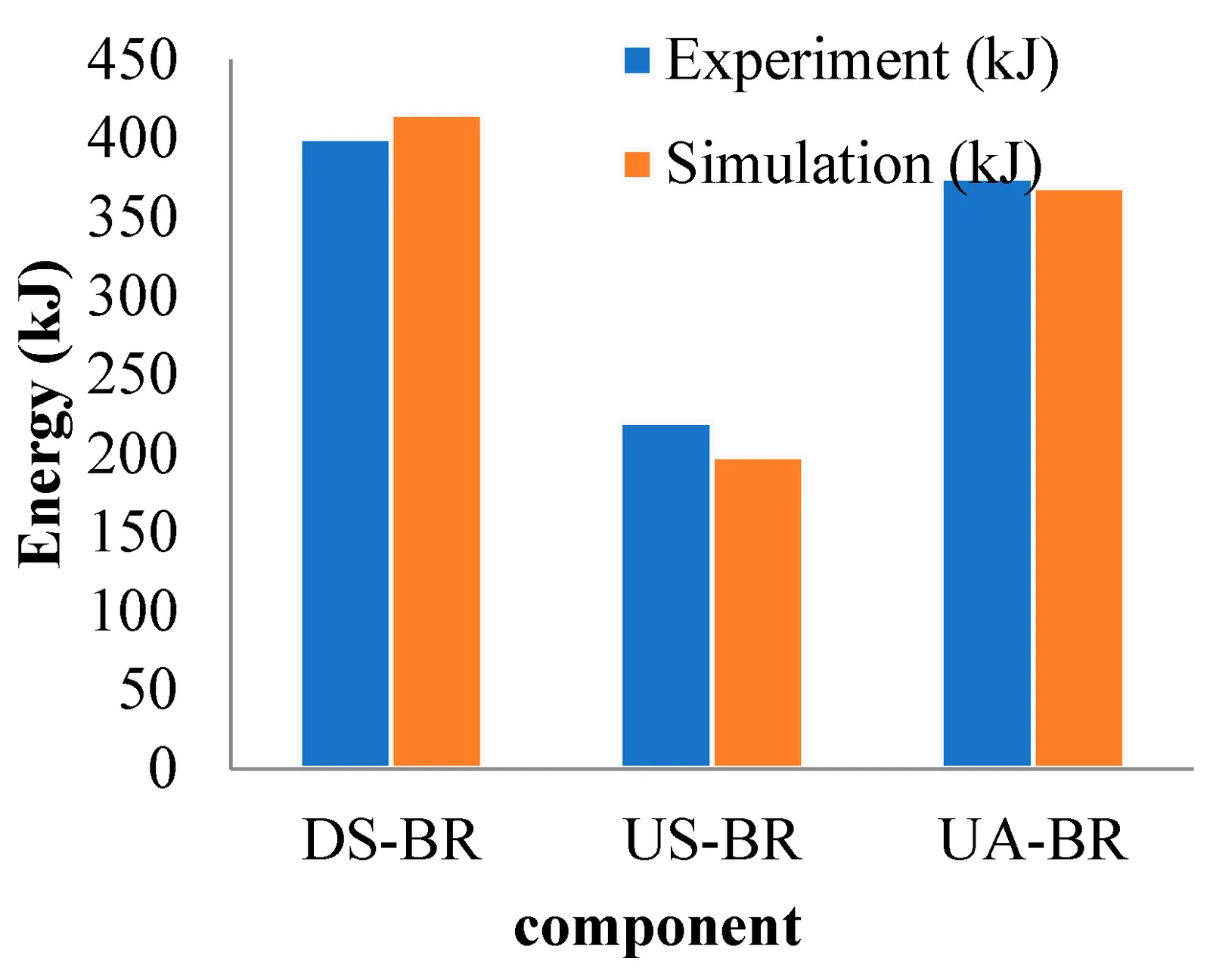
Comparison of the energy dissipation of each part of the pressure-relief rings.

**Table 1:** Specimen specifications.

Specimen No.	Net Type	Wire Diameter (mm)	Ring Diameter (mm)	Tension Specimen Size (mm)	Puncture Specimen Size (mm)
R8-3.0-300	R8	3	300	1100 × 1100	3200 × 3200
R9-3.0-300	R9	3	300	1100 × 1100	3200 × 3200
R12-3.0-300	R12	3	300	1100 × 1100	3200 × 3200
R16-3.0-300	R16	3	300	1100 × 1100	3200 × 3200
R19-3.0-300	R19	3	300	1100 × 1100	3200 × 3200

**Table 2:** Tension test results.

Specification	Breaking Force (kN)	Peak Displacement (mm)
R8-3.0-300	240.63 ± 10.87	333.76 ± 7.28
R9-3.0-300	251.31 ± 6.14	359.87 ± 26.09
R12-3.0-300	459.30 ± 42.99	343.22 ± 12.09
R16-3.0-300	547.89 ± 70.42	331.02 ± 15.85
R19-3.0-300	611.22 ± 25.81	348.98 ± 57.37

**Table 3:** Puncture test results.

Specification No.	Breaking Force (kN)	Peak Displacement (mm)
R8-3.0-300	423.37 ± 102.90	1100.18 ± 40.55
R9-3.0-300	654.03 ± 69.56	1125.67 ± 24.87
R12-3.0-300	958.51 ± 34.38	1135.14 ± 17.02
R16-3.0-300	1247.80 ± 14.69	1138.27 ± 27.47
R19-3.0-300	1501.79 ± 127.13	1164.87 ± 10.60

**Table 4:** Comparison of the unified piecewise constitutive model parameters for the tension and puncture cases.

Parameter	Tension Case	Puncture Case	Physical Meaning	Case Difference
Tensioning-segment stiffness *k*_0_	8.75	42.4	kN per unit displacement	Puncture tensioning stiffness is about 4.8 times that of tension
Power-hardening exponent *p*	6.51	5.78	—	Tension hardening is steeper
End of tensioning segment *χ*_1_	0.097	0.026	—	Puncture tensioning segment is shorter
Peak displacement *χ*_max_	0.313	0.227	—	Puncture peak displacement is smaller
Peak force per steel wire *F*_max_	32.54	33.4	kN	The two cases are close
Bearing capacity–specification relationship	*F* = 35.3*n* − 30	*F* = 38.8*n* − 84.9	kN	Unilateral puncture value is comparable to that of tension
Model *R*^2^	0.98	0.983	—	—

**Table 5:** Structural configuration of the full-scale impact test flexible rockfall barrier.

Member	Configuration
Upper support rope	Two high-strength steel wire ropes, 22 mm in diameter, each equipped with three pressure-relief rings at both ends
Lower support rope	Same configuration as the upper support rope
Upper anchor rope	One high-strength steel wire rope, 22 mm in diameter, with two pressure-relief rings in series
Pressure-relief ring	Single pressure-relief ring calibrated to dissipate 50 kJ

**Table 6:** Equivalent geometric and material parameters of the equivalent element of the R12-3.0-300 ring net.

Equivalent Element Parameter	Tension Case	Puncture Case
Steel wire diameter *d*_0_ (mm)	3.0	3.0
Number of wound wires *n*	12	12
Ring radius *R* (mm)	150	150
Equivalent section radius *r*_truss_ (mm)	28.27	28.27
Equivalent section area *A*_eq_ (mm^2^)	2511.5	2511.5
Initial length of equivalent element (mm)	150	150
Material density *ρ* (kg/m^3^)	7850	7850
Initial modulus of the tensioning segment *E*_t0_ (MPa)	5.70	2.15
Poisson’s ratio *ν*	0.3	0.3
Stiffness of tensioning segment *k*_0_ (kN)	8.75	42.4
Hardening exponent *p*	6.51	5.78
Displacement at end of tensioning segment *χ*_1_	0.097	0.026
Peak displacement *χ*_max_	0.313	0.227
Peak force *F*_max_ (kN)	32.54	33.4

Note: the equivalent element adopts the two-stage σ–ε constitutive law established in Section 3; its modulus is not a fixed value, but is defined by the tensioning-segment initial modulus *E*_t0_ (fixed) followed by a power-hardening segment whose tangent modulus increases with strain. The complete response is defined by the parameters *k*_0_, *p*, *χ*_1_, *χ*_max_ and *F*_max_ listed above together with the equivalent geometry.

**Table 7:** Material parameters of the members in the overall structure.

Member	Element Type	Density (kg/m^3^)	Poisson’s Ratio *ν*	Young’s Modulus *E* (MPa)	Yield Stress (MPa)
Steel-wire rope (support rope/anchor rope)	Cable	7900	0.3	1.5 × 10^5^	1770
Supporting steel column	User-defined beam element	7900	0.3	2.0 × 10^5^	235

**Table 8:** Contact types and friction settings.

Contact Pair	Contact Type	Algorithm	Friction Coefficient *M*
Ring net—support rope	AUTOMATIC_SURFACE_TO_SURFACE	Penalty	0.1
Support rope—anchor point	Guided-cable contact	Penalty	0.1
Ring net—impact block	AUTOMATIC_SURFACE_TO_SURFACE	Penalty	0.1
Ring net self-contact	AUTOMATIC_SINGLE_SURFACE	Penalty	0.1

**Table 9:** Element type.

Member	Ring Net (Side Span, Tension)	Ring Net (Impact Span, Puncture)	Support Rope/Anchor Rope	Pressure-Relief Ring	Supporting Steel Column
Element type	Truss	Truss	Cable	Energy-dissipating	Beam

**Table 10:** Comparison of the energy dissipation of the pressure-relief rings.

Member	Test Energy Dissipation (kJ)	Simulation Energy Dissipation (kJ)	Relative Error
Lower support-rope pressure-relief rings	400.0	415.2	+3.8%
Upper support-rope pressure-relief rings	220.0	198.1	−10.0%
Upper anchor-rope pressure-relief rings	375.0	368.8	−1.7%

## Data Availability

The original contributions presented in this study are included in the article. Further inquiries can be directed to the corresponding author.

## References

[1] Ge Z., Liu H. (2022). Numerical investigation on the dynamic response of rockfall impacts on flexible barrier under seismic loading. Nat. Hazards.

[2] Li X., Zhao J., Kwan J.S.H. (2020). Assessing debris flow impact on flexible ring net barrier: A coupled CFD-DEM study. Comput. Geotech..

[3] Zhao L., Zhang L., Yu Z., Qi X., Xu H., Zhang Y. (2022). A case study on the energy capacity of a flexible rockfall barrier in resisting landslide debris. Forests.

[4] Volkwein A., Schellenberg K., Labiouse V., Agliardi F., Berger F., Bourrier F., Dorren L.K.A., Gerber W., Jaboyedoff M. (2011). Rockfall characterisation and structural protection: A review. Nat. Hazards Earth Syst. Sci..

[5] Zhu Z.H., Yin J.H., Qin J.Q., Tan D.Y. (2019). A new discrete element model for simulating a flexible ring net barrier under rockfall impact comparing with large-scale physical model test data. Comput. Geotech..

[6] Nicot F., Cambou B., Mazzoleni G. (2001). From a constitutive modelling of metallic rings to the design of rockfall restraining nets. Int. J. Numer. Anal. Methods Geomech..

[7] Zhao Y.N., Yu Z.X., Zhao S.C. (2019). Zoned equivalent model of ring interception nets in flexible protection systems. J. Southwest Jiaotong Univ..

[8] Coulibaly J.B., Chanut M.A., Lambert S., Nicot F. (2019). Toward a generic computational approach for flexible rockfall barrier modeling. Rock Mech. Rock Eng..

[9] Gentilini C., Gottardi G., Govoni L., Mentani A., Ubertini F. (2013). Design of falling rock protection barriers using numerical models. Eng. Struct..

[10] Sautter K., Hofmann H., Wendeler C., Wilson P., Bucher P., Bletzinger K.-U., Wüchner R. (2021). Advanced modeling and simulation of rockfall attenuator barriers via partitioned DEM-FEM coupling. Front. Built Environ..

[11] Guo L.P., Yu Z.X., Luo L.R., Qi X., Zhao S.C. (2020). Analytical method for the puncture mechanical behavior of ring nets based on force-flow equivalence. Eng. Mech..

[12] Jin Y.T., Yu Z.X., Guo L.P., Luo L.R., Zhang L.J., Liao L.X. (2023). Tension-bending stiffness coordination model for strand ring nets in flexible rockfall protection systems. Chin. J. Rock Mech. Eng..

[13] Jin Y., Yu Z., Guo L., Zhang L., Liao L., Luo L. (2024). Failure behavior and bearing capacity prediction of steel wire-ring nets under rockfall impact using a coordination model-based analysis method. Eng. Fail. Anal..

[14] Qi X., Deng Q., Zhao L., Yuan S., Li Z., Wang X., Yu Z. (2023). Equivalent relationship of the mechanical behavior of ring nets under static punching and dynamic impact conditions. Buildings.

[15] Trad A., Limam A. (2022). Load testing and simulation of asymmetric cable-net used for flexible rockfall protection barriers. Eur. J. Environ. Civ. Eng..

[16] Castanon-Jano L., Blanco-Fernandez E., Castro-Fresno D. (2019). Design of a new energy dissipating device and verification for use in rockfall protection barriers. Eng. Struct..

[17] He Z.H., Yu Z.X., Liao L.X., Lyu Y.F., Tian Y.D. (2024). Research on data-driven intelligent design method for energy dissipator of flexible protection systems. Adv. Steel Constr..

[18] Qi X., Yu Z., Zhao L., Xu H., Zhao S. (2018). A new numerical modelling approach for flexible rockfall protection barriers based on failure modes. Adv. Steel Constr..

[19] Xia C., Zhang Z., Liu C., Zhang H., Tian S. (2022). Study on numerical model and dynamic response of ring net in flexible rockfall barriers. Sustainability.

[20] Singer V., Larese A., Börst A., Wüchner R., Bletzinger K.U. Partitioned MPM-FEM Coupling Approach for Advanced Numerical Simulation of Mass-Movement Hazards Impacting Flexible Protective Structures. Proceedings of the 10th International Conference on Computational Methods for Coupled Problems in Science and Engineering (COUPLED PROBLEMS 2023).

[21] Debelak A.M., Bareither C.A., Mahmoud H. (2020). Finite-element model of impact loading and deformation of a flexible steel ring-net debris-flow barrier. Nat. Hazards Rev..

[22] Liu C., Yu Z., Zhao S. (2020). Quantifying the impac of a debris avalanche against a flexible barrier by coupled DEM-FEM analyses. Landslides.

[23] Pimpinella F., Marchelli M., De Biagi V. (2024). A weight-based efficiency measure for energy dissipating devices for flexible rockfall barriers. Int. J. Prot. Struct..

[24] Zhao L., Zou D.F., Qi X., Ma P.C. (2025). Load transfer mechanisms in support ropes of flexible rockfall barriers: Geometrical relationships and transmission attenuation. Int. J. Prot. Struct..

[25] (2020). Flexible Protection Net System of Slope.

[26] Guo L., Yu Z., Jin Y., Liao L., Luo L. (2022). An analytical method for evaluating the deflection and load-bearing and energy absorption capacity of rockfall ring nets considering multifactor influence. Adv. Steel Constr..

[27] Zhao Y.N., Yu Z.X., Zhao S.C. (2019). Numerical calculation method for multi-span flexible passive ring-net barrier structures. J. Vib. Shock.

[28] Zhu Z.H., Yin J.H., Ouyang C.J., Tan D.Y., Qin J.Q. (2020). Modeling a flexible ring net with the discrete element method. J. Eng. Mech..

[29] Jin Y., Yu Z., Guo L., Liao L., Zhang L., Luo L. (2025). An efficient hybrid simulation method to quantitatively analyze the flexible wire-ring net rockfall protection systems. Int. J. Numer. Anal. Methods Geomech..

[30] Liao L., Yu Z., Liu D., Luo L., Guo L., Tian X. (2023). Design method and impact response of energy-consuming high-fall flexible protection system for construction. Buildings.

[31] Yu Z., Zhao L., Guo L., Liu Y.P., Yang C., Zhao S.C. (2019). Full-scale impact test and numerical simulation of a new-type resilient rock-shed flexible buffer structure. Shock Vib..

[32] Koo R.C.H., Kwan J.S.H., Lam C., Ng C.W.W., Yiu J., Choi C.E., Ng A.K.L., Ho K.K.S., Pun W.K. (2017). Dynamic response of flexible rockfall barriers under different loading geometries. Landslides.

[33] Qi X., Zhao L., Meng Q.C. (2024). Prediction method for the tension force of support ropes in flexible rockfall barriers based on full-scale experiments and numerical analysis. Sci. Rep..

[34] Guo L., He S., Yu Z., Jin Y. (2025). Damage assessment of ring nets in flexible barriers subjected to consecutive rockfall impacts. Can. Geotech. J..

[35] Lambert S., Toe D., Mentani A., Bourrier F. (2021). A meta-model-based procedure for quantifying the on-site efficiency of rockfall barriers. Rock Mech. Rock Eng..

